# The Impact of Door to Diuretic Time in Acute Heart Failure on Hospital Length of Stay and In-Patient Mortality

**DOI:** 10.7759/cureus.12742

**Published:** 2021-01-16

**Authors:** Arshad Muhammad Iqbal, Sohaib K Mohammed, Nida Zubair, Ateeq Mubarik, Adnan Ahmed, Syed Farrukh Jamal, Syed Moin Hassan, Furqan Haq, Salman Muddassir

**Affiliations:** 1 Internal Medicine, University of South Florida Morsani College of Medicine Graduate Medical Education (GME) Oak Hill Hospital, Brooksville, USA; 2 Cardiology/Electrophysiology, University of Missouri School of Medicine, Columbia, USA; 3 Internal Medicine, Deccan College of Medical Sciences, Hyderabad, IND; 4 Internal Medicine, Dow Medical College and Civil Hospital, Karachi, PAK; 5 Internal Medicine, Oak Hill Hospital, Brooksville, USA; 6 Sleep Medicine, New York Sleep Disorder Center, Brooksville, USA; 7 Internal Medicine, Ascension St. Michael's Hospital, Stevens Point, USA; 8 Internal Medicine, Saint Joseph Hospital, Chicago, USA; 9 Cardiology, Cleveland Clinic Abu Dhabi, Abu Dhabi, ARE; 10 Internal Medicine, North Shore Medical Center, Salem, USA; 11 Miscellaneous, Hospital Corporation of America West Florida, Tampa, USA

**Keywords:** door to diuretic time, mortality, acute decompensated heart failure, length of hospital stay (los)

## Abstract

Background

Acute heart failure (AHF) can be life-threatening if not treated promptly and can significantly increase the number of annual emergency department (ED) encounters in the United States. Achieving adequate and prompt euvolemic state in AHF patients using intravenous (IV) diuretics is the cornerstone of treatment, which not only reduces in-hospital stay and mortality but also decreases healthcare expenditures. Surprisingly, the door to diuretic (D2D) time in AHF patients has always been a debatable issue among physicians worldwide, and so far, there are no set guidelines. This study examines a large cohort of AHF patients to determine the association between diuretics use within 90 minutes of ED admission and hospital length of stay (LOS) and patient mortality.

Methods

Retrospective institutional data of AHF patients receiving IV diuretics following ED admission were extracted from 2016 to 2017. A total of 7,751 patients treated for AHF exacerbation were included, which were further divided into two groups based on the timing of diuretics administration (<90 minutes vs. ≥90 minutes). The primary outcomes were LOS between the two groups and hospital mortality. The standard statistical methodology was used for data analysis.

Results

A total of 7,751 AHF cases receiving IV diuretics were identified. Almost 1,432 patients (18.5%) received IV diuretics within 90 minutes of ER admission (group 1) while 6,319 patients (81.5%) patients received IV diuretics after 90 minutes (group 2). Furthermore, among group 1 patients, average LOS was noted to be associated with shorter hospitalization (average of 1.423 days less as compared to group 2 patients (confidence interval (CI) =1.02-1.82; p<0.05). Finally, after controlling for other mortality risk factors, patients in group 2 were 1.435 times more likely to have died compared to patients in group 1 (CI=1.03-1.98; p<0.05).

Conclusions

D2D time in AHF patients has always been a crucial judgmental decision. The current study successfully demonstrated the relation between IV diuretics administration within 90 minutes of ED admission, favorable clinical outcomes, and decreased mortality rates. More adequately powered studies are needed to validate the results of our current study further.

## Introduction

In the United States (US), more than five million adults suffer from heart failure (HF), and almost half of the patients diagnosed with HF die within five years of diagnosis [1]. Acute heart failure (AHF) is the rapid onset or worsening of symptoms and/signs of HF and is a life-threatening condition with high morbidity and mortality rates [2-3]. A majority of AHF patients are evaluated and managed in the emergency department (ED) [2]. More than 80% of these patients are admitted to the hospital with approximately 3.4 days median inpatient length of stay (LOS). Over the last decade, the mortality rate remained high despite a slight decline in the hospital stay rate [4-5].

For several decades, the management of AHF has not changed, and the majority of drug trials were unsuccessful in demonstrating a favorable prognostic impact [2]. Intravenous (IV) diuretics remain the mainstay for the treatment of AHF [6]. Early treatment with IV furosemide has been independently associated with better in-hospital mortality (IHM) rates [7]. The door to diuretic (D2D) time in AHF patients has always been a debatable issue among physicians worldwide, and so far, there are no set guidelines. This retrospective study examines a large cohort of AHF patients to determine the association of diuretic use within 90 minutes of ED admission with hospital LOS and in-hospital mortality.

The abstract of this article has been presented at the American College of Cardiology Conference in March 2019 [8].

## Materials and methods

Study design and setting

A large retrospective cohort of a total of 7,751 adult patients (≥18 years) treated for AHF exacerbation and receiving IV diuretics (such as furosemide or bumetanide) following ED admission was extracted from 2016 to 2017, which was further divided into two groups based on the timing of diuretic administration (Group 1 <90 minutes vs. Group 2 ≥90 minutes). Data were retrieved by using the International Classification of Diseases 10th Revision (ICD-10) coding from 16 urban hospitals in West Florida.

Demographics and clinical factors

We included patients aged 18 years and above admitted following ED admission with AHF exacerbation. Only data of their first visit were used. The data were extracted using the institutional enterprise data warehouse (EDW)

Data collection

The medical records of all patients admitted with AHF at the 16 aforementioned hospitals in West Florida were extracted on a Health Insurance Portability and Accountability Act (HIPPA)-compliant, password-restricted computer with limited access to researchers only. We used ICD-10 codes to extract data from the EDW. The primary outcomes were LOS between the two groups and in-hospital mortality.

Statistical analysis

Descriptive data were provided as mean and standard deviations. Multiple regression analysis was used to predict the length of stay (LOS) based on the age, sex, patient’s brain natriuretic peptide (BNP; pg/mL) and troponin (ng/mL) levels, intravenous (IV), and type of diuretics. Logistic regression was performed to determine the odds of being a case based on the predictors of mortality during the length of stay.

Data aggregates were created for the length of stay and door to diuretics time of <90 minutes and ≥90 minutes. Pearson correlation was used to determine the correlation between the door to diuretics and length of stay (LOS). A p-value of 0.05 was considered to be statistically significant. A Kaplan Meier survival analysis was performed to determine the time for door to diuretics for furosemide and bumetanide. We performed all statistical analyses with the Statistical Package for the Social Sciences (SPSS; Version 23.0., IBM Corp., Armonk, NY).

## Results

A total of 7,751 AHF cases receiving IV diuretics were identified. Almost 1,432 patients (18.5%) received IV diuretics within 90 minutes of ED admission (group 1) while 6,319 patients (81.5%) patients received IV diuretics after 90 minutes (group 2). Furthermore, among group 1 patients, average LOS was noted to be associated with a shorter hospitalization (average of 1.423 days less as compared to group 2 patients (confidence interval (CI)=1.02-1.82; p<0.05). Finally, after controlling for other mortality risk factors, patients in group 2 were 1.435 times more likely to have died as compared to patients in group 1 (CI=1.03-1.98; p<0.05). Table 1 shows the group statistics of patients who died during hospitalization with no significant difference in mean with respect to age, sex, IV diuretic, and type of diuretic (bumetanide vs furosemide).

**Table 1:** General statistics for independent variables: showing patients who died during hospitalization with no significant difference in mean with respect to age, sex, IV diuretic, and type of diuretic (bumetanide vs furosemide) IV: intravenous; BNP: brain natriuretic peptide

Characteristics	Death	N	Mean	Std. Deviation
Age (yrs)	No	7424	67.11	10.31
	Yes	327	70.43	8.48
Sex	No	7424	.57	.49
	Yes	327	.57	.49
BNP (pg/mL)	No	7424	6480.68	12749.36
	Yes	327	11564.78	17932.86
Troponin (ng/mL)	No	7424	.51	9.25
	Yes	327	1.34	6.09
IV	No	7424	.85	.36
	Yes	327	.91	.28
Bumetanide (mg)	No	7424	.11	.31
	Yes	327	.14	.35
Furosemide (mg)	No	7424	.81	.38
	Yes	327	.86	.34

The average age of the patients was 67.1±10.31 in this study. Sex (p=0.837) and type of diuretics (p=0.88) were found not to be statistically significant. Patients who did not receive their diuretic within 90 minutes were 1.435 times (p=0.029) more likely to have died controlling for the other factors as compared to counterparts who did receive their diuretic within 90 minutes. Table 2 shows the logistic regression of death during stay with no significant difference in sex and type of diuretic use.

**Table 2:** Logistic regression for death during stay with no significant difference in sex and type of diuretic use a. Variable(s) entered on step 1: diu_ind2, Age in yrs, sex_ind, lg10_bnp, lg10_trop, route1, diu_type01. SlowDiuretic: 0 if diuretic in first 90 minutes, 1 if more; -Sex_ind: 0 for female, 1 for male; -log10(BNP): Log base 10 of BNP (pg/mL); -log10(Trop): Log base 10 of Troponin (ng/mL); -IV_Ind: 0 for PO, 1 for IV; -Bumetanide_Ind: 0 for Furosemide, 1 for Bumetanide IV: intravenous; BNP: brain natriuretic peptide

Variables in the Equation									
		B	S.E.	Wald	df	Sig.	Exp(B)	95% C.I. for EXP (B)	
								Lower	Upper
Step 1^a^	SlowDiuretic_Ind	.361	.165	4.779	1	.029	1.435	1.038	1.984
	Age	.037	.007	29.622	1	.000	1.038	1.024	1.052
	Sex_Ind	-.024	.116	.042	1	.837	.976	.777	1.227
	log_10_(BNP)	.294	.095	9.527	1	.002	1.342	1.113	1.617
	log_10_(Trop)	.671	.066	101.759	1	.000	1.955	1.717	2.228
	IV_Ind	.542	.197	7.534	1	.006	1.719	1.168	2.531
	Bumetanide_Ind	.283	.166	2.903	1	.088	1.327	.958	1.838
	Constant	-6.697	.630	113.152	1	.000	.001		

If a diuretic was not given in the first 90 minutes, it correlates to a 1.423 (p<0.005) day longer length of stay. Table 3 shows multiple regression for the length of stay with a significant difference in mean with respect to duration of diuretic administration, sex, BNP, troponin, and type of diuretics.

**Table 3:** Multiple regression for the length of stay with a significant difference in the mean with respect to the duration of diuretic administration, sex, BNP, troponin, and type of diuretics -SlowDiuretic: 0 if diuretic in first 90 minutes, 1 if more; -Sex_ind: 0 for female, 1 for male; -log10(BNP): Log base 10 of BNP (pg/mL); -log10(Trop): Log base 10 of Troponin (ng/mL); -IV_Ind: 0 for PO, 1 for IV; -Bumetanide_Ind: 0 for Furosemide, 1 for Bumetanide IV: intravenous; BNP: brain natriuretic peptide

Coefficients^a^								
Model		Unstandardized Coefficients		Standardized Coefficients	t	Sig.	95.0% Confidence Interval for B	
		B	Std. Error	Beta			Lower Bound	Upper Bound
1	(Constant)	4.754	.736		6.458	.000	3.311	6.196
	SlowDiuretic_Ind	1.423	.205	.079	6.956	.000	1.022	1.824
	Age	.000	.008	.000	-.018	.986	-.015	.015
	Sex_Ind	-.756	.159	-.054	-4.753	.000	-1.068	-.444
	log_10_(BNP)	.462	.127	.043	3.642	.000	.213	.710
	log_10_(Trop)	.682	.087	.092	7.824	.000	.512	.853
	IV_Ind	1.089	.220	.056	4.942	.000	.657	1.521
	Bumetanide_Ind	1.197	.253	.053	4.730	.000	.701	1.693

**Figure 1:**
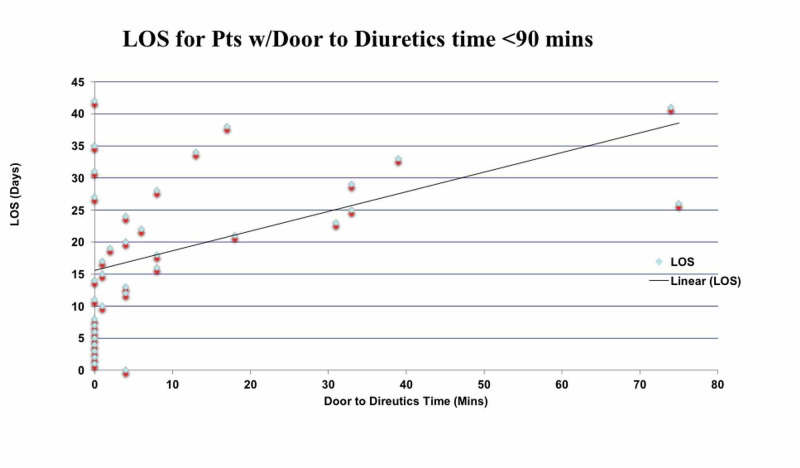
Correlation between length of stay of patient (Y-axis) with door to diuretics time of < 90 minutes (X-axis)

**Figure 2:**
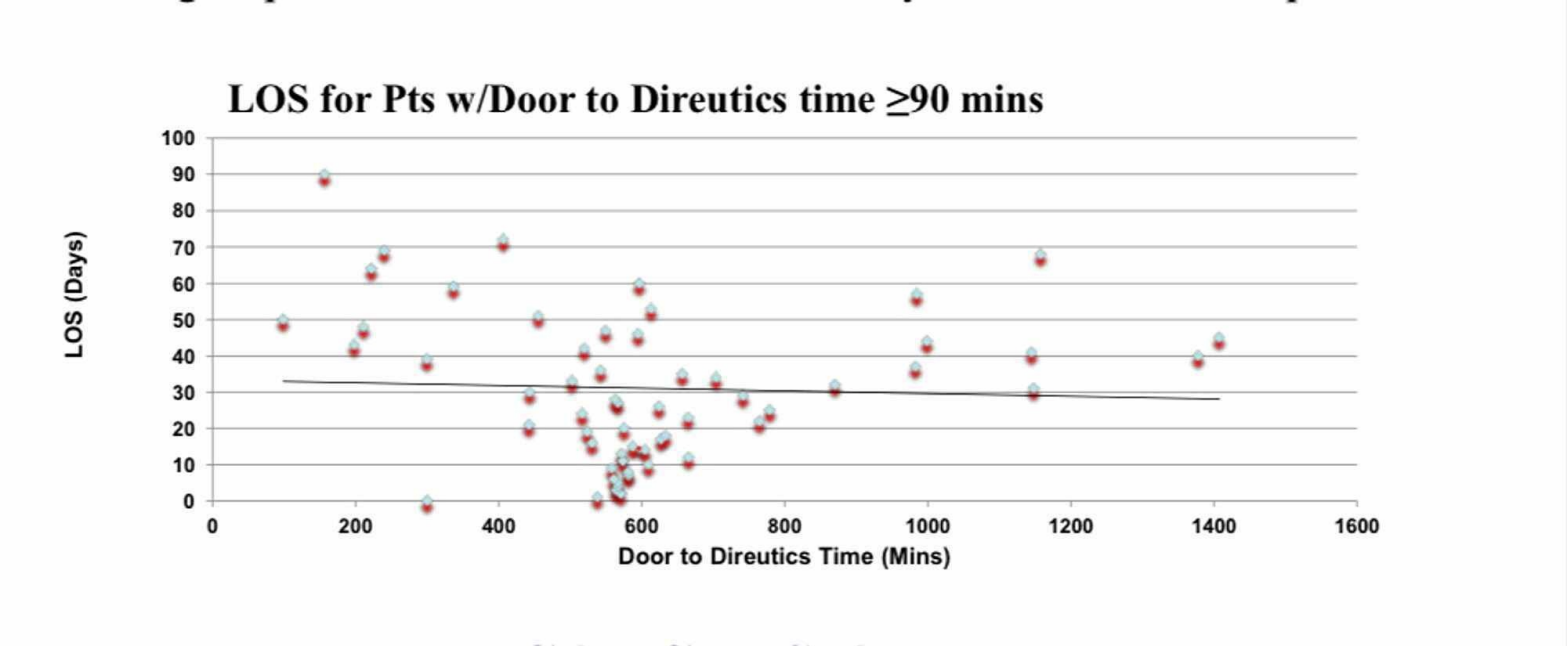
Correlation between length of stay of patient (Y-axis) with door to diuretics time of ≥ 90 minutes (X-axis)

**Figure 3:**
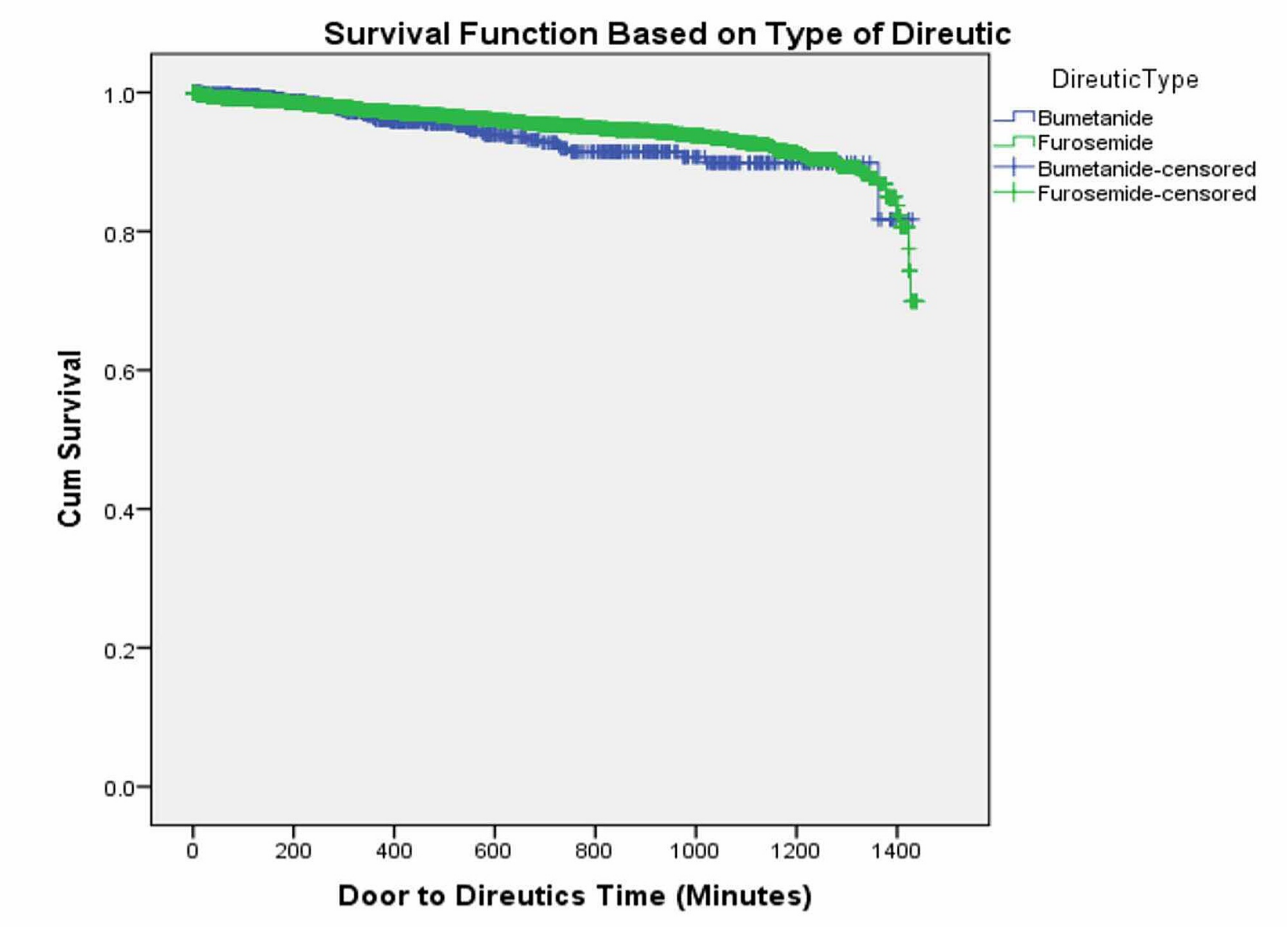
Survival distribution of patients receiving bumetanide vs furosemide

A positive correlation, r=0.50, p<0.05, was found between length of stay and door to diuretic time of <90 minutes (Figure 1) while a very weak negative correlation, r=-0.048, p=0.72, was found between length of stay and door to diuretic time of ≥90 minutes (Figure 2). The Kaplan Meier curves' last cumulative survival proportion demonstrates that the proportion of patients that had furosemide was not different from those on bumetanide (Figure 3). A log-rank test showed that there is no difference in the overall survival distributions between furosemide and bumetanide (p=0.15) (Figure 3).

## Discussion

AHF is the rapid onset or exacerbation of heart failure, characterized by the signs and symptoms of fluid overload, which warrants immediate medical attention and intervention [9-10]. Patients may present with AHF as the first presentation of heart disease but more commonly as decompensation of pre-existing cardiomyopathy. In the latter case, admission to a hospital represents a significant prognostic event in the natural history of cardiomyopathy, as it is associated with worsening outcomes. Despite many advances in medical therapies, AHF has very rates of morbidity and mortality [10].

Diuretics are the mainstay for the treatment of AHF [6]. Early and rapid treatment in the ED is an important step in management [11]. Several studies have substantiated the positive effect of early treatment on the prognosis of patients with AHF, and the delay was associated with a significant increase in the risk of in-hospital mortality (IHM) [7]. Maisel et al.'s retrospective study demonstrated that for every four-hour delay in time to first IV furosemide, there is a 2.1% increase in the IHM [12]. Matsue et al. in their prospective study substantiated that increase in the door to furosemide time steeply increases the mortality risk to the first 100 minutes, and this effect is leveled off thereafter. Early treatment of AHF patients with IV furosemide is independently associated with a decrease in IHM. These might support the current recommended window time of 30 to 60 minutes for IV furosemide after ED arrival in AHF patients [13].

However, Park et al. concluded from their prospective trial on the Korean population that the door to diuretic time has no impact on the IHM, post-discharge one-month and one-year mortality [14].

In our retrospective observational study, we demonstrated that the time for diuretics infusion in AHF patients does affect clinical outcomes. Patients treated with diuretics within 90 minutes of ED presentation had better clinical outcomes and lower mortality rate as compared to patients who received IV diuretics after 90 minutes. Moreover, hospital LOS was more in the latter group. There is a moderately strong correlation between hospital length of stay and door to diuretics time of <90 minutes while no correlation was found for ≥90 minutes in this large retrospective study. Door to diuretics less than 90 minutes is a predictor of better outcomes of shorter length of stay and reduced mortality. The type of diuretic did not have any effect on patient survival.

The importance and definitive effect of early diuretic administrations on clinical outcomes need to be investigated more in the future by prospective randomized controlled clinical trials.

The major limitation of our study is that it is a retrospective study, which may have an element of information and recall bias.

## Conclusions

D2D time in AFH patients has always been a crucial judgmental decision. The current study successfully demonstrated the relation between IV diuretics administration within 90 minutes of ED admission, favorable clinical outcomes, and decreased mortality rates. More prospective randomized control studies are needed to validate the results of our current study.
